# RAC1 signaling in prostate cancer: VAV GEFs take center stage

**DOI:** 10.3389/fcell.2025.1658639

**Published:** 2025-08-15

**Authors:** Mariana Cooke, Nahuel Peinetti, Marcelo G. Kazanietz, Kerry L. Burnstein

**Affiliations:** Department of Molecular and Cellular Pharmacology, University of Miami Miller School of Medicine and Sylvester Comprehensive Cancer Center, Miami, FL, United States

**Keywords:** Vav2, Vav3, Rac1, EGFR, prostate cancer, migration, cancer signaling

## Introduction

In a recent study, Baker et al. (2025) identified the Rac guanine nucleotide exchange factor VAV2 as a marker of poor prognosis and a signaling link that contributes to the proliferation and aggressiveness of castration-resistant prostate cancer (CRPC) cells. Prostate cancer (PCa) is the most common noncutaneous malignancy among men worldwide, with 1 in 8 men diagnosed with this disease during their lifetime. While patients with organ-confined, locally advanced, or regionally spread disease display a 5-year survival rate greater than 99%, the survival rate for advanced-stage disease with distant metastatic spread declines to 30%–40% (American Cancer Society, 2025). Androgen deprivation therapy (ADT) remains the cornerstone of treatment for patients with high-risk localized and advanced PCa. Despite initial biochemical or radiological remission after ADT, most patients eventually progress to metastatic castration-resistant prostate cancer (mCRPC), a highly heterogeneous, aggressive, and lethal disease. Deregulation of oncogenic and invasive signaling pathways represents a major hallmark of CRPC cells, enabling their escape from the primary tumor to secondary sites, particularly the axial skeleton (Rebello et al., 2021).

Rac1, a member of the Rho GTPase family, represents a crucially deregulated signaling player leading to tumor progression, particularly in the metastatic spread of cancer cells. Rac1 has been recognized as a major regulator of actin cytoskeleton reorganization, which promotes the formation of cell surface projections (e.g., lamellipodia, membrane ruffles) necessary for cell migration and invasion during metastasis. Additionally, Rac1 regulates a diverse range of cellular functions in cancer cells, including proliferation, gene expression, metabolism, and epithelial-to-mesenchymal transition (EMT), making it an attractive target for cancer therapy (Bustelo, 2018; Kazanietz and Caloca, 2017; De et al., 2020; Casado-Medrano et al., 2019). Like most members of the Rho GTPase family, Rac1 functions as a binary switch, being active in its GTP-bound form and inactive in its GDP-bound form. This “on-off” cycling is tightly regulated by Rac Guanine nucleotide Exchange Factors (Rac-GEFs), which facilitate GTP loading and thus activate Rac1. Inactivation of Rac1 is mediated by GTPase-activating proteins (Rac-GAPs) that accelerate GTP hydrolysis. Active (GTP-bound) Rac1 relays through various effectors, triggering a complex network of signaling events that influence both actin dynamics and diverse cellular processes independent of actin cytoskeleton remodeling. Extracellular cues, such as those involving ligand-mediated stimulation of receptor tyrosine kinases (RTKs) and G protein-coupled receptors (GPCRs), represent the most common upstream inputs that confer Rac1 activation (Bustelo, 2018; Kazanietz and Caloca, 2017; Kazanietz et al., 2022). The large size of the Rac-GEF family, which comprises 32 Dbl-like and 11 DOCK Rac-GEFs, along with their distinctive expression based on cell type (Casado-Medrano et al., 2018), suggests multifaceted coupling mechanisms that depend on the nature of the receptor and Rac-GEF, resulting in the activation of discrete intracellular Rac1 pools and exquisite selectivity for downstream responses. Mechanistically, the diversity of Rac-GEF/Rac1 signaling likely relies on strict spatiotemporal regulation of Rac-GEFs by specific receptors and their coupling to effectors (e.g., PI3K), ultimately influencing downstream responses through a complex modulation of the Rac1 interactome (Kazanietz et al., 2022; Banka et al., 2022).

## Rac-GEF signaling in prostate cancer: identification of VAV2 as an RTK effector

Rac1 is often deregulated in pathological conditions, including neurological diseases and cancer (Bustelo, 2018; Kazanietz and Caloca, 2017; De et al., 2020; Casado-Medrano et al., 2019; Casado-Medrano et al., 2018; Banka et al., 2022). While cutaneous melanoma can harbor activated Rac1 mutants, this is rare (Krauthammer et al., 2012). Instead, Rac1 deregulation is due to abnormally elevated Rac-GEF expression or hyperactivation of receptors that promote Rac-GEF activation (Bustelo, 2018; Kazanietz and Caloca, 2017; Casado-Medrano et al., 2018). In PCa, constitutively elevated Rac1 activity has been observed in several cellular models of androgen receptor (AR) negative PCa, including DU145, PC3, and PC3-ML cell lines, compared to normal prostate epithelial cells or androgen-dependent PCa cells (Baker et al., 2020). In their recent study, Baker et al. (2025) demonstrated that Rac1 deficiency leads to significant defects in the migratory and proliferative capacities of CRPC cellular models. The migratory defect aligns with the expected role of Rac1 in actin cytoskeleton-dependent motility and invasion signaling. Furthermore, it correlates strongly with bioinformatics analysis in the TCGA-PRAD human prostate carcinoma database, which reveals worse progression-free survival in PCa patients with signatures predicting “high Rac1 cell motility activity.” Rac1 deficiency also leads to significant changes in gene expression, particularly affecting transcriptional networks related to cell adhesion, ECM functions, migration, proliferation, and inflammation. Despite the negative regulation of E-cadherin expression by Rac1, the loss of Rac1 was insufficient to reverse the mesenchymal phenotype typical of AR-null PCa cells.

Identifying the GEF(s) responsible for Rac activation in any given model is daunting due to limited knowledge about the spatial and temporal expression of individual members of the large Rac-GEF family and their activation statuses. Overcoming this challenge is critical to assigning specific functional roles to individual Rac-GEFs in processes associated with oncogenesis and metastasis. Using a pre-designed Q-PCR array, Baker et al. (2025) defined the Rac-GEF mRNA abundance in both castration-resistant and androgen-dependent PCa cell lines. This analysis revealed a relatively common expression pattern among the two groups and a shared subset of Rac-GEFs compared to cell lines derived from other cancer types, namely, adrenocortical and lung cancer (Cooke et al., 2023; Cooke et al., 2021). ECT2, TRIO, FARP1, PLEKHG2, VAV2, PREX1, and FARP2 were identified as the top-expressed Dbl-like Rac-GEFs in PCa cells, while DOCK1, DOCK5, DOCK7, and DOCK9 were the top-expressed DOCK family Rac-GEFs. Through the use of the PARADIGM algorithm, statistically significant positive correlations were identified between the expression of discrete Rac-GEFs and the “Rac1 cell motility pathway,” with the highest correlation found for the Rac-GEF VAV2 (p = 6.7 × 10^−10^). Functional studies using VAV2-deficient DU145 PCa cells established this Rac-GEF as a key cell migration and proliferation driver. Interestingly, RNAi screening revealed VAV2 to be the only Rac-GEF capable of driving Rac1 activation in response to ligand-mediated stimulation of EGFR (Baker et al., 2025), an RTK with established roles in PCa progression, including metastatic dissemination (Day et al., 2017). VAV2 was also found to mediate the invasiveness of PCa cells (Cooke et al., manuscript in preparation).

## Aberrantly elevated VAV expression in human prostate cancer

**FIGURE 1:**
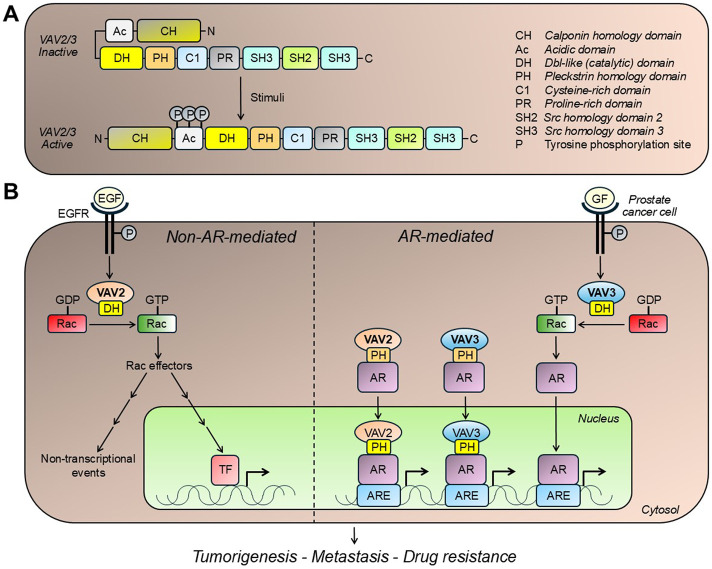
VAV isoforms and prostate cancer. **(A)** Structure of VAV isoforms expressed in prostate cancer. VAV2 and VAV3 activation occurs upon stimulation of receptor tyrosine kinases, such as EGFR. This process involves a conformational rearrangement that exposes the DH catalytic domain, as well as domains implicated in lipid interactions (e.g., PH and C1 domains) and protein interactions (e.g., SH2 and SH3 domains). **(B)** VAV isoforms mediate effects in prostate cancer cells both in androgen receptor (AR)-independent and -dependent manners. VAV2 and VAV3 promote GDP/GTP exchange on Rac1, the main Rac small GTPase expressed in prostate cancer cells. This small G-protein has been widely implicated in proliferative and migratory signaling, therefore contributing to prostate tumorigenesis and metastasis. VAV isoforms also enhance ligand-independent AR nuclear translocation and transcriptional activity, contributing to the proliferative and tumorigenic capacities of prostate cancer cells. See text for details. ARE, androgen receptor response element; EGF, epidermal growth factor; EGFR, epidermal growth factor receptor; GF, growth factor; P, phosphorylation; TF, transcription factor.

The mammalian VAV family of Rac-GEFs comprises three members: VAV1, VAV2, and VAV3 (Bustelo, 2014). According to mRNA expression, VAV2 is the most highly expressed VAV isoform in PCa cells, followed by VAV3 (Figure 1A). In contrast, VAV1, which is primarily expressed in hematopoietic cells, is essentially undetectable in PCa cell lines (Baker et al., 2025). Baker et al. conducted an immunohistochemical analysis using a large number of human PCa specimens, establishing prominent upregulation of VAV2 in tumoral areas compared to non-tumoral areas. No significant VAV2 staining could be observed in the prostate stroma, ruling out the possibility of microenvironmental effects of VAV2 in PCa progression. These results were strongly supported by bioinformatic analysis of databases, including TCGA-PRAD, which shows VAV2 as the top upregulated Rac-GEF in PCa compared to normal tissue. Database analysis also revealed the progressive upregulation of VAV2 with increasing Gleason score, as well as in metastasis (Baker et al., 2025), in agreement with Magani et al. (2017). Kaplan-Meier analysis revealed VAV2 to be a negative predictor for disease-specific survival (DSS), disease-free interval (DFI), and progression-free interval (PFI), underscoring the potential prognostic value of this Rac-GEF in human PCa (Baker et al., 2025). Despite VAV2 being the most highly expressed VAV isoform in PCa, studies have also revealed that VAV3 levels are upregulated during the *in vivo* progression of PCa cell lines to castration resistance (Lyons and Burnstein, 2006; Lin et al., 2012; Lyons et al., 2008). VAV3 expression is elevated in late-stage and metastatic PCa, and its expression in early-stage tumors is associated with a lower overall biochemical failure-free survival rate (Lin et al., 2012). Notably, its expression as a transgene in mouse prostates leads to the development of prostatic intraepithelial neoplasia (PIN) and PCa (Liu et al., 2008). Similar to VAV2, VAV3 has been established as an EGFR effector and can mediate Rac1 activation in response to EphA2 RTK stimulation (Lin et al., 2012). Therefore, it is plausible that both VAV isoforms may participate in PCa progression. Since VAV2 and VAV3 are structurally related, possible functional redundancy may occur in prostate cancer, although co-expression of these VAV isoforms in human prostate tumors has not been thoroughly investigated. Nonetheless, unique non-redundant roles for VAV isoforms have also been described (Pearce et al., 2004; Conde et al., 2021; Fujikawa et al., 2003). The reported upregulation in VAV3 expression and activation observed in VAV1/VAV2-deficient models suggests the existence of compensatory mechanisms controlling VAV isoform expression and is indicative of their complex functional interdependence (Chang et al., 2012). The availability of genetically engineered VAV2/VAV3 mouse models (Pearce et al., 2004; Conde et al., 2021; Fujikawa et al., 2003; Chang et al., 2012; Sauzeau et al., 2007; Quevedo et al., 2010; Menacho-Márquez et al., 2013) would be instrumental in establishing unique and/or distinctive roles in prostate cancer progression *in vivo*.

## VAV isoforms: roles in androgen receptor function and drug resistance

Research from our group and others demonstrated a complex crosstalk between VAV family members and AR in PCa (Magani et al., 2017; Lyons and Burnstein, 2006; Dong et al., 2006; Peacock et al., 2012; Rao et al., 2012) (Figure 1B). Through its GEF activity, VAV3 can trigger Rac1 signaling, enhancing ligand-independent AR nuclear translocation and transcriptional activity, which in turn increases PCa cell proliferation (Lyons et al., 2008). Alternatively, both VAV2 and VAV3 can act independently of their GEF activity and serve as co-activators of full-length AR and constitutively active AR splice variants (e.g., AR-V7) (Wu et al., 2013; Wang et al., 2025). This AR coactivation is mediated by direct binding to AR through the DH domain as well as by binding to AR co-chaperones such as Cdc31, and possibly SRC-1 and SRC-2 (Magani et al., 2017; Wu et al., 2013). The PH domain has been shown to promote AR N/C interactions, leading to nuclear translocation and the formation of a transcriptional complex that regulates AR target gene expression (Magani et al., 2017; Rao et al., 2012). This role as a coactivator has been linked to CRPC progression (Peacock et al., 2012; Rao et al., 2012). Despite the well-defined differences in the mechanisms of action by which VAVs activate AR in PCa, it remains unclear whether one can prevail over the others during disease progression. While publicly available patient data sets show a correlation of VAV2 and VAV3 with AR-V7 in bone metastatic CRPC (Magani et al., 2017), the interplay between these two VAV isoforms and AR activity is another crucial aspect that requires further elucidation.

With the rise of advanced targeted and hormone-based therapies, overcoming therapeutic resistance has become a primary challenge in PCa management. Increased expression of AR coactivators has been identified as a mechanism by which PCa escapes AR-targeted therapies. Recently, binding of VAV2 to AR and AR splice variants has been shown to stabilize these receptors and mediate enzalutamide resistance (Wang et al., 2025). Additionally, genetically engineered cells with reduced expression levels of VAV3 exhibit an improved response to docetaxel in preclinical models of PCa (Nomura et al., 2013). Disrupting the interaction between the DH domain of VAV3 and the TAU5 region of AR using protein fragments decreased AR-V7 nuclear localization and, as a result, reduced cell proliferation and migration while increasing apoptosis, thus demonstrating the clinical relevance of targeting VAVs in PCa (Magani et al., 2017). Direct targeting of VAVs poses a challenge - as is also the case for most Rho-family GEFs - since these molecules are subject to intricate regulatory mechanisms (i.e., phosphorylation, protein-protein interactions) and lack druggable pockets for selective pharmacological targeting (Neurath and Berg, 2024; Smithers and Overduin, 2016). Drug discovery efforts exploiting unique interfaces involved in GEF/GTPase interactions have led to the development of promising antitumor and antimetastatic small-molecule inhibitors (Bustelo, 2018; Kazanietz and Caloca, 2017). A notable example that highlights the strong feasibility for the design of inhibitors of VAV-Rac/Cdc42 interactions is the development of Ehop-016 and Ehop-097 (Montalvo-Ortiz et al., 2012; Medina et al., 2022). Ehop-016 shows excellent pharmacological activity in mouse models of experimental metastasis with no significant toxicity (Castillo-Pichardo et al., 2014; Humphries-Bickley et al., 2015). Proof-of-principle for the potent anti-migratory activity of Ehop-097 in CRPC cells has been established in Baker et al. (2025). Recently, Nassar and coworkers identified IODVA1 as a first-in-class small-molecule VAV3 inhibitor, likely acting by locking this GEF into an autoinhibitory state that prevents Rac access to the DH catalytic domain (Hegde et al., 2022). With the development of new small-molecule inhibitors for VAVs, preclinical testing efforts will be crucial in determining the translational potential of VAV inhibitors in PCa.

## Concluding remarks

Vav family members represent key therapy-resistant nodes in advanced PCa (AR and non-AR expressing) and serve as potential biomarkers of poor clinical outcomes. As GEFs for Rac1, these proteins relay diverse oncogenic signals and control crucial steps in the metastatic dissemination process. VAV2 and VAV3 coactivate AR, a primary driver of PCa, promoting proliferation and therapy resistance. The enhancement of AR activity by VAVs can occur in a GEF-independent manner, posing a unique therapeutic challenge. Developing specific VAV inhibitors that can be utilized in distinct clinical settings alongside patient-risk stratification would be instrumental in advancing PCa therapeutic strategies. Although PCa treatment has made significant strides, new resistance mechanisms have emerged, including tumors with neuroendocrine features as well as “double negative” tumors that lack both AR expression and neuroendocrine markers. Given the fact that VAVs can promote both AR-dependent and AR-independent growth, targeting VAV/Rac signaling pathways offers a novel and promising approach for enhancing PCa management.
